# Understanding the complexities of mathematical cognition: A multi-level framework

**DOI:** 10.1177/17470218231175325

**Published:** 2023-05-27

**Authors:** Camilla Gilmore

**Affiliations:** Centre for Mathematical Cognition, Loughborough University, Loughborough, UK

**Keywords:** Mathematical cognition, mathematics education, numerical processing, executive functions, spatial skills, language

## Abstract

Mathematics skills are associated with future employment, well-being, and quality of life. However, many adults and children fail to learn the mathematics skills they require. To improve this situation, we need to have a better understanding of the processes of learning and performing mathematics. Over the past two decades, there has been a substantial growth in psychological research focusing on mathematics. However, to make further progress, we need to pay greater attention to the nature of, and multiple elements involved in, mathematical cognition. Mathematics is not a single construct; rather, overall mathematics achievement is comprised of proficiency with specific components of mathematics (e.g., number fact knowledge, algebraic thinking), which in turn recruit basic mathematical processes (e.g., magnitude comparison, pattern recognition). General cognitive skills and different learning experiences influence the development of each component of mathematics as well as the links between them. Here, I propose and provide evidence for a framework that structures how these components of mathematics fit together. This framework allows us to make sense of the proliferation of empirical findings concerning influences on mathematical cognition and can guide the questions we ask, identifying where we are missing both research evidence and models of specific mechanisms.

Mathematics skills are associated with future employment, well-being, and quality of life (Crawford & Cribb, 2013; Gerardi et al., 2013; Reyna et al., 2009). However, many children and adults struggle with learning mathematics. For example, in the United Kingdom, a fifth of children do not achieve the expected standard by the end of primary school (Department for Education, 2019), and almost half of adults fail to acquire the mathematical skills needed to succeed in modern life (Organisation for Economic Co-Operation and Development, 2013). Disadvantage gaps in mathematics are large, emerge early, and are resistant to intervention (Andrews et al., 2017; Aubrey et al., 2006; Aunio & Niemivirta, 2010; Crenna-Jennings, 2018). Moreover, many adults and children have negative associations or anxiety about mathematics, even if they achieve at the expected levels (Dowker et al., 2016).

To remedy this situation, it is clear, therefore, that there is a need to improve mathematics education. To do this effectively, we need to have a better understanding of the processes of learning and performing mathematics. This goes beyond questions about pedagogy, of interest only to educational researchers; understanding mathematical cognition raises deeper questions that are of relevance to psychologists. For example, mathematical cognition research addresses questions such as how uniquely human systems of symbolic representation integrate with evolutionarily old, intuitive systems for representing mathematical ideas; or how affective processes interact with domain-general and domain-specific cognitive mechanisms.

There has been a substantial growth in research taking a psychological perspective on the mechanisms and processes involved in mathematics learning over the past two decades (Gilmore et al., 2018; Inglis & Foster, 2018). To make sense of this increasing literature and develop improved theory necessary to inform mathematics education, we must pay greater attention to the nature of mathematical cognition and the multiple elements involved. Below, I first consider the multi-componential nature of mathematics before proposing a framework that provides a structure by which we can reconcile the proliferation of empirical findings concerning the influences on mathematical cognition. It makes explicit assumptions about the nature of mathematical cognition that are often not expressed and exposes the elements of mathematics that receive less research attention. It can guide the questions we ask and identify where we are missing both research evidence and models of mechanisms. I then highlight how examples of existing research map onto the framework, before considering the unanswered questions and implications.

## What is mathematics?

Before we can develop a framework concerning the processes and influences on mathematical cognition, it is necessary to first consider what we mean by mathematics. Too often, research in mathematical cognition has taken a narrow view of what constitutes mathematics (Alcock et al., 2016) with a focus only on arithmetical procedural fluency (i.e., the speed and accuracy with which individuals can complete written or mental abstract arithmetic) or number fact knowledge (i.e., the retrieval of number facts without using procedural mental strategies). However, mathematics is a multi-componential, not a unitary, construct, and we will fail to make progress in understanding the breadth of influences on mathematical cognition if we continue with this narrow focus. Moreover, our research will be of less interest and relevance to educators who take a broader perspective.

If we look beyond arithmetical procedures and facts, then there are different ways in which we can categorise mathematics. First, we can take what might be considered a curricular view by distinguishing between different domains of mathematics (e.g., arithmetic, geometry, statistics, algebra). This approach separates mathematics according to the nature of the mathematical information. An alternative is to distinguish different types of knowledge (e.g., Hiebert & Lefevre, 1986; Rittle-Johnson et al., 2015). For example, a distinction is often made between factual knowledge (i.e., *knowing that*), procedural skills (i.e., *knowing how*), and conceptual understanding (i.e., *knowing why*; Bisanz & LeFevre, 1990). This distinction can be applied within each curricular mathematical domain. These broad distinctions between domains and types of knowledge begin to reveal the multi-componential nature of mathematics. However, neither of these approaches may be sufficient to identify the mechanisms and cognitive processes involved in mathematics performance because these are still broad categories.

**Figure 1.**
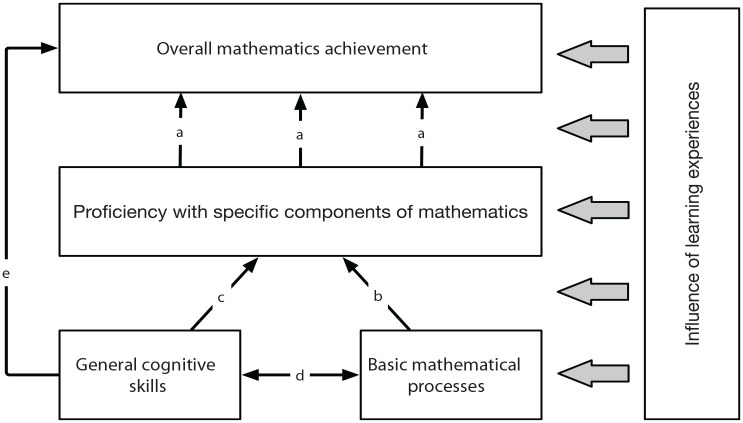
A multi-level framework of mathematical cognition. According to the framework, overall mathematics achievement emerges from proficiency with specific components of mathematics, which in turn recruit basic mathematical processes. General cognitive skills are independently related to basic mathematical processes, proficiency with specific components of mathematics and overall mathematical achievement. Informal and formal learning experiences may influence the development of each level of the framework, as well as the links between them. Evidence for each of the links a – e can be found in the text.

To gain greater understanding of the mechanisms and processes involved in learning and performing mathematics, it may therefore be helpful to distinguish three levels of mathematical cognition (Figure 1). First, *overall mathematics achievement* is an individual’s overall attainment in mathematics. It is typically measured by broad curriculum measures or composite standardised measures that incorporate a variety of areas of mathematics and may include reasoning, problem solving, and contextual mathematical problems. Mathematics achievement requires more than just competency with a set of individual skills. Measures of mathematics achievement typically require individuals to identify the mathematics required in contextually based problems to select appropriate strategies and to combine different skills and knowledge to answer a given question. Doing this successfully is likely to require an understanding of how different components of mathematics relate to each other and the ability to integrate new knowledge with existing knowledge and skills.

Second, *proficiency with specific components of mathematics* captures an individual’s performance in coherent sub-components of mathematics for which it may be anticipated that they will use a more-or-less consistent set of mathematical knowledge and skills. For example, specific components of mathematics may include number fact retrieval, algebraic reasoning, understanding of arithmetical relationships, and adaptive strategy selection (see Table 1 for examples). The nature and content of these components may change over development and learning, and it is possible to define these components more or less narrowly. What should drive our selection of these components for research studies is to consider how useful these subdivisions are for the aims of our research. For example, sometimes it may be appropriate to consider procedural arithmetic skills as a single component, but other times, it may be appropriate to consider addition, subtraction, multiplication, and division as separate components, depending on the question being answered and the age of the participants.

**Table 1.** Examples of specific components of mathematics and basic mathematical processes that have been the focus of previous research.

Specific components of mathematics	Basic mathematical processes
Count sequence knowledge Number fact fluency Mental arithmetic Written arithmetic procedures Understanding of arithmetical relationships Word-problem solving Adaptive strategy selection Algebraic thinking Solving algebraic word problems Composition of shapes	Single-digit number comparison Multi-digit number comparison Non-symbolic magnitude comparison Number line estimation Numerical order processing Spatial-numerical associations Place-value understanding Intuitive geometrical knowledge Pattern recognition Analogical reasoning

Finally, there are *basic mathematical processes*, which are lower-level processes that underpin the specific components described above. Here, it is helpful to consider the lowest levels of mathematical processes that cannot be easily subdivided and measured in a meaningful (mathematical) fashion. This might include magnitude comparison, order processing, spatial-numerical associations, intuitive geometrical knowledge, and place-value understanding. The nature of these basic mathematical processes may change over development and learning and again the selection of these for use in research studies should be driven by specific research questions. For example, sometimes, it may be appropriate to consider symbolic magnitude comparison holistically, but other times, it may be appropriate to distinguish between single-digit and multi-digit symbolic magnitude comparison.

## A multi-level framework for mathematical cognition

Taking a multi-level, multi-component view of mathematics may be helpful for understanding the mechanisms of mathematical cognition and the influences on them. For example, over the past two decades, a growing body of research has identified that mathematics performance is related to a set of cognitive skills (see De Smedt, 2022, for a review). Much of this research has considered “mathematics” as a unitary construct, either explicitly by using broad measures of mathematics achievement, or implicitly by treating measures of proficiency with specific components of mathematics (e.g., number fact retrieval, written procedural fluency) as synonymous with mathematics achievement more generally. These alternative approaches each raise potential issues: (1) use of broad measures of mathematics achievement, which incorporate a range of topics and require proficiency with a range of specific components of mathematics, makes it difficult to identify the specific mechanisms by which cognitive (or non-cognitive) factors may play a role in learning or performing mathematics; and (2) using measures of proficiency with specific components of mathematics as synonymous with mathematics achievement makes it difficult to compare and combine findings across different studies. For example, we would not expect the cognitive skills involved in retrieving number facts to be the same as the cognitive skills involved in performing a written algorithm for multi-digit multiplication. Hence, use of different mathematics measures may be one of the sources of conflicting findings in the literature.

Thinking about the multiple levels of mathematics outlined above (overall achievement, proficiency with specific components, basic processes) can help us to see how existing empirical findings fit together and identify where future research effort should be focused. This approach also encourages greater attention to be paid to the complexity and multi-componential nature of mathematics. According to the framework (Figure 1), overall mathematics achievement emerges from proficiency with a set of specific components of mathematics, which in turn recruit basic mathematical processes. Domain-general cognitive skills can have separate influences at each of these levels (i.e., domain-general skills may operate independently at each level). The characteristics and opportunities afforded by children’s informal and formal learning experiences affect mathematical learning and processes, and this influence can occur at each of the levels or on the links between them.

The proposed framework is not a model of mathematical processes; however, it can help us identify the particular mathematical processes that we wish to understand. The links between different elements of the framework represent mechanisms. Theoretical models already exist that provide more detail about some of these specific mechanisms. For example, the Pathways model (LeFevre et al., 2010) proposes a set of processes which explain how some specific mathematical components (e.g., calculation) arise from certain domain-general skills (language, spatial skills) and basic mathematical processes (subitising); Cragg, Keeble, et al. (2017) proposed a model to explain how different executive function skills are associated with proficiency with different specific mathematics components as well as overall mathematics achievement; Hawes and Ansari (2020) proposed a set of mechanisms to explain the association between spatial skills and components of mathematics (including number ordering, word-problem solving, and written arithmetic); Cipora et al. (2020) considered specific mechanisms by which spatial processes are recruited in basic mathematical processes; and Wong (2021) proposed a set of specific processes related to mathematical problem solving. These models focus on particular mechanisms, which is essential for our understanding of mathematical cognition, but they do not provide a structure for how these mechanisms fit within a broader view of achievement in mathematics.

In the following section, I consider each of the links (*a–e*) in the framework and briefly review current evidence about the nature and importance of each link. A full review of the literature is beyond the scope of this article, but the examples given are intended to provide a flavour of the existing research.

## Existing evidence for the multi-level framework of mathematical cognition

### (a) The connection between proficiency with specific components of mathematics and overall mathematical achievement

That there is an association between proficiency with specific components of mathematics and overall mathematical achievement is likely the most self-evident connection within the framework. But it is important, methodologically, theoretically, and pedagogically, not to conflate these levels. Individual differences in overall mathematics achievement do not inform us, per se, about individual differences in proficiency with specific components of mathematics. Moreover, components of mathematics are not simply hierarchical whereby an individual’s proficiency with one component directly determines their proficiency with another component in a simplistic fashion.

The complex relationship between proficiency with different components of mathematics has been recognised for some time. Dowker (2015) highlighted that there can be discrepancies in an individuals’ proficiency with specific components such as counting and word-problem solving. This has been particularly investigated in relation to children’s conceptual and procedural knowledge of arithmetic, with evidence that children’s procedural arithmetic skills are not a good indicator of their level of conceptual understanding (Canobi, 2004; Gilmore & Papadatou-Pastou, 2009). Similar discrepancies have been observed between exact calculation skills and computational estimation (Dowker, 2005).

Due to the componential nature of mathematics, an individual’s overall level of mathematics achievement might not be a good indicator of their proficiency with specific components of mathematics. For example, we measured 5- to 6-year-old children’s overall mathematics achievement as well as proficiency with specific arithmetic skills (Gilmore et al., 2017). We found that children could be meaningfully clustered according to their procedural arithmetic skills, conceptual understanding of arithmetic, and working memory. However, clusters of children with very different profiles of performance across these components could not be distinguished using only their performance on a measure of overall mathematics achievement.

Given that overall mathematics achievement is separable from proficiency with specific components of mathematics, questions arise concerning which specific components are (most strongly) associated with mathematics achievement, and what else determines individual difference in mathematics achievement. Many studies have investigated the components of mathematics that are predictors of concurrent or future mathematics achievement. Overall mathematics achievement is associated with number fact retrieval, written and mental procedural arithmetic skills, and understanding of arithmetic principles (e.g., Cowan et al., 2011; Cragg, Keeble, et al., 2017; Geary et al., 2017; Nunes et al., 2012). Consistent evidence also suggests that understanding of fractions is associated with concurrent and future mathematics achievement (e.g., Bailey et al., 2012; Siegler et al., 2012; Torbeyns et al., 2015). The constellation of mathematics components that are associated with overall mathematics achievement changes over development and education, with evidence that symbolic number knowledge is a key competency in the early years of schooling (e.g., Jordan et al., 2009). In support of claims that algebra is a “gatekeeper” topic for future success in mathematics and STEM (science, technology, engineering, and mathematics) subjects more broadly, algebra has been found to be strongly associated with mathematics achievement in adolescence (Trickett et al., 2022).

The specific components of mathematics that have received most attention from mathematical cognition research have typically focused on knowledge (e.g., number fact knowledge) or skills (e.g., performance of arithmetic procedures). Less research has focused on conceptual understanding, that is, individuals’ understanding of the principles and relationships that underlie a specific domain (Hiebert & Lefevre, 1986; Rittle-Johnson et al., 2015). And yet conceptual understanding may be a more important predictor of overall mathematics achievement than procedural measures. Developing good conceptual understanding allows learners to understand why procedures must be followed in certain ways, to identify efficient strategies, to apply their knowledge and skills appropriately in novel situations, and to avoid errors that stem from misconceptions. The importance of deep conceptual understanding is recognised by educators. Although pockets of research on conceptual understanding from a mathematical-cognition perspective do exist (e.g., understanding of rational numbers, Hallett et al., 2010; understanding of arithmetic principles, Robinson & Dubé, 2009; understanding the cardinality principle, Geary et al., 2018), it is by-and-large under-studied in comparison with mathematical knowledge and skills (Alcock et al., 2016). One reason for this is likely to be that measuring conceptual understanding is more challenging than measuring children’s skills or knowledge. However, methods for measuring conceptual understanding do exist (e.g., Bisson et al., 2016; Crooks & Alibali, 2014). To identify the most important specific components that relate to overall mathematics achievement, we need to pay more attention to conceptual understanding. Research needs to measure what is important and not just what is easy to measure.

### (b) The connection between basic mathematical processes and proficiency with specific components of mathematics

Specific components of mathematics recruit a set of more basic mathematical processes. Over the past two decades, there has been a notable increase in studies investigating the nature of these basic processes. Most of this work has considered basic processes in the numerical domain (e.g., numerical magnitude processing, numerical order processing), but there are some exceptions (e.g., intuitive geometry, logical reasoning). Interpreting this evidence in the light of the multi-component framework is not straightforward because these studies often use measures of overall mathematics achievement and proficiency with specific components of mathematics interchangeably. Here I focus on evidence for associations with proficiency with specific components of mathematics rather than overall mathematics achievement. Where associations have been found with measures of overall mathematics achievement, these tend to be interpreted via mechanisms that act on proficiency with specific components of mathematics.

A considerable amount of attention has been paid to basic magnitude comparison processes, both symbolic (i.e., comparing pairs of digits) and non-symbolic (i.e., comparing dot arrays). Although findings vary across studies, meta-analyses suggest that there is a reliable relationship between these processes and proficiency with specific components of mathematics, which is stronger for symbolic comparison processes than non-symbolic comparison processes. Schneider et al. (2017) found that magnitude comparison skills were associated with mental and written arithmetic. There was also an association with curriculum measures of mathematics, but this was weaker than the relationship with proficiency with more specific components of mathematics. In individual studies, specific relationships have been found between magnitude comparison skills and arithmetic strategy selection (Vanbinst et al., 2012), mental arithmetic (Linsen et al., 2015), written multi-digit arithmetic (Linsen et al., 2015), approximate calculation skills (Gunderson & Hildebrand, 2021), and geometry (Lourenco et al., 2012).

Number line tasks are used as an alternative measure of numerical magnitude processing. Again, meta-analyses provide evidence for reliable relationships between number line task performance and counting, mental arithmetic, and written arithmetic (Schneider et al., 2018). In contrast to magnitude comparison, the relationship with overall mathematical achievement measures was equivalent to the relationship with proficiency with more specific components. In individual studies, specific relationships have been found between number line tasks and mental arithmetic (Laski & Yu, 2014), written calculation (LeFevre et al., 2013), timed arithmetic procedures (Link et al., 2014), understanding of fractions (Hansen et al., 2015), approximate calculation skills (Gunderson & Hildebrand, 2021), count sequence knowledge (Muldoon et al., 2013), and geometry (McDougal et al., 2022).

Numerical ordering skills (e.g., recognising that triplets of digits are either in numerical order or not in numerical order) have received increasing attention (see Devlin et al., 2022, for a review). Specific associations with ordering skills have been found for written abstract arithmetic (Goffin & Ansari, 2016), timed measures of arithmetic fluency (Lyons & Ansari, 2015; Morsanyi et al., 2017; Sasanguie et al., 2017), and count sequence knowledge (Gilmore & Batchelor, 2021).

Aside from numerical skills, other proposed basic mathematical processes include patterning skills and intuitive geometry. Research on patterning skills is relatively recent and much has focused on preschoolers. Patterning skills are associated with verbal calculation (Zippert et al., 2019), calculation skills (Fyfe et al., 2017), composite numeracy skills (Wijns et al., 2019), and problem solving (Di Lonardo Burr et al., 2022), and also predicts growth in early mathematics achievement (Rittle-Johnson et al., 2019). Intuitive geometry skills (i.e., untaught basic geometrical processing, Spelke et al., 2010) are associated with academic geometry performance (Giofrè et al., 2013). However, we do not yet know to what extent this is a basic mathematical process separable from spatial skills.

Given that many basic mathematical processes involve domain-general cognitive skills, and these cognitive skills are also associated with proficiency with specific components of mathematics components (see below), it is important to test how far general cognitive skills might explain the relationship between basic mathematical processes and proficiency with specific components of mathematics (or vice versa). While in many cases, the associations between basic mathematical processes and proficiency with specific components of mathematics remain after controlling for cognitive skills (e.g., Fuchs, Geary, Compton, Fuchs, Hamlett, & Bryant, 2010; Mazzocco et al., 2011; O’Connor et al., 2018), there are some exceptions. Several studies have demonstrated that the relationship between non-symbolic magnitude comparison and mathematics outcomes is explained by inhibitory control (Fuhs & McNeil, 2013; Gilmore et al., 2013; Keller & Libertus, 2015; but see Malone et al., 2019). Similarly, Simms et al. (2016) found that visuospatial skills explained the relationship between linearity estimates from a number line task and mathematics achievement. Therefore, to understand the specific mechanisms of mathematics learning, it is important to consider both specific mathematical processes and more general cognitive skills.

### (c) The connection between general cognitive skills and proficiency with specific components of mathematics

There is a growing body of correlational and experimental evidence demonstrating associations between general cognitive skills and proficiency with specific components of mathematics. A wide range of cognitive skills have been investigated; as examples, I here consider evidence related to working memory, inhibitory control, spatial skills, and language skills. This evidence comes from studies with participants from pre-school to adults. While some studies have considered their role in basic mathematical processes (see section (d)) or association with overall mathematics achievement (see section (e)), it is important to also consider how they relate to proficiency with specific components of mathematics, such as number fact knowledge or algebraic thinking. This matters because it is likely that different sets of domain-general skills are related to different components of mathematics (Cirino et al., 2016; Cragg, Keeble, et al., 2017; Fuchs, Geary, Compton, Fuchs, Hamlett, Seethaler, et al., 2010; Träff et al., 2018). These relationships may encompass more direct mechanisms than the association between domain-general skills and mathematics achievement more generally. Therefore, uncovering these mechanisms is necessary to build causal models of mathematical cognition.

We now have a large body of evidence concerning the association between executive function skills and specific components of mathematics. However, most of these studies have focused on procedural measures of arithmetic (although they may draw conclusions about mathematics more generally). Meta-analysis has identified that working memory is most strongly related to whole-number calculation and word-problem solving, with a weaker relationship with geometry (Peng et al., 2016). This meta-analysis considers only rather broad components of mathematics, but individual studies have found a relationship between working memory and proficiency with specific components of mathematics, for example, arithmetic fluency (Van de Weijer-Bergsma et al., 2015), multi-digit written arithmetic (Fuchs, Geary, Compton, Fuchs, Hamlett, Seethaler, et al., 2010), fraction computation (Hecht et al., 2003), number fact knowledge (Cragg, Keeble, et al., 2017), understanding of arithmetic concepts (Andersson, 2010), and algebraic word problems (Lee et al., 2009). Dual-task studies have also identified that working memory is directly implicated in number fact retrieval (Cragg, Richardson, et al., 2017), and the use of procedural arithmetic strategies (Imbo et al., 2005; Imbo & Vandierendonck, 2007).

Beyond working memory, relationships have also been found between inhibitory control and proficiency with specific components of mathematics. For example, children’s inhibitory control is associated with adaptive strategy selection (Lemaire & Lecacheur, 2011), counting proficiency (Lan et al., 2011), number fact retrieval (Cragg, Keeble, et al., 2017), rational number processing (Abreu-Mendoza et al., 2018), and use of conceptually based arithmetic strategies (Eaves et al., 2022; Robinson & Dubé, 2013). Experimental approaches have also provided supporting evidence for a role of inhibitory control in number fact retrieval (De Visscher & Noël, 2014; Eaves et al., 2022; Megías et al., 2015). Some evidence points to an association between cognitive flexibility and proficiency with specific components of mathematics (e.g., calculation; Andersson, 2010), although fewer studies have investigated this, and the evidence is more mixed than that focusing on inhibition (Santana et al., 2022; Yeniad et al., 2013).

Turning to spatial skills, meta-analysis has established an overall reliable association between spatial reasoning and mathematics achievement (Xie et al., 2020) that is stronger for logical reasoning (considered to also include problem solving) than numerical or arithmetical skills. However, this analysis only distinguished between fairly broad domains of mathematics. There is both experimental and associational evidence for the involvement of spatial skills in a range of specific mathematics components. Factor analytic studies have identified associations between spatial skills and proficiency with specific mathematics components (e.g., place value, word-problem solving, calculation, fraction processing and algebra; Mix et al., 2016). Students who make use of spatial strategies have been found to have higher accuracy on word problems (Boonen et al., 2013), spatial skills are related to procedural arithmetic skills and geometry (McDougal et al., 2022), and concurrent calculation skills (although not growth in calculation skills; Gunderson & Hildebrand, 2021). Experimental studies have highlighted how manipulating spatial characteristics can affect mathematical processes, for example, spatial grouping effects have been found for arithmetic calculation (e.g., Landy et al., 2014; Landy & Goldstone, 2010). Positive effects of spatial training have been found for a range of mathematical outcomes, including calculation (Cheng & Mix, 2014; see Hawes et al., 2022, for a review). These studies demonstrate a causal link between spatial skills and mathematics. However, the precise mechanisms underlying such a relationship and the extent and stability of training effects need to be determined before the educational relevance of spatial training is established.

Finally, linguistic influences can be observed on many aspects of mathematics (see Dowker & Nuerk, 2016, for overview). While much of this evidence has focused on either basic mathematical processes or overall mathematics achievement (see below), some studies have pinpointed relationships between language and proficiency with more specific components of mathematics. For example, children’s language skills (including vocabulary and phonological skills) are related to early numerical skills in preschool children (Cahoon et al., 2021; LeFevre et al., 2010; Purpura et al., 2011). In school-aged children, language skills are associated with arithmetic skills (Träff et al., 2018; Vukovic & Lesaux, 2013), and language comprehension is associated with word-problem solving (Fuchs et al., 2006). Word-problem solving has been consistently related to language skills (see Daroczy et al., 2015, for a review).

Additional evidence for the importance of language skills on proficiency with specific mathematics components comes from evidence of linguistic differences. Languages differ in the transparency of number naming, that is, whether the number naming systems clearly map onto the base-10 system. In particular, some languages such as German have the inversion property, where two-digit numbers above 20 are named with the units first (e.g., 42 is named “zweiundvierzig” which translates to “two-and-forty”), and this affects mathematics skills. For example, specific differences in single-digit arithmetic are observed between those who speak languages that do or do not include the inversion property (Van Rinsveld et al., 2015), and differences between more or less transparent languages (e.g., Chinese vs. English) have been observed on counting skills (Mark & Dowker, 2015). In both these studies, the linguistic influences were found to be specific to certain mathematics skills but not others, highlighting the importance of considering proficiency with specific mathematics components and not just overall mathematics achievement.

### (d) The connection between general cognitive skills and basic mathematical processes

In addition to the correlational and experimental evidence regarding the associations between general cognitive skills and proficiency with specific components of mathematics, there is also evidence that general cognitive skills are implicated in basic mathematical processes (e.g., Hohol et al., 2017). First, associations have been reported between working memory measures and the processing of symbolic and nonsymbolic numerical quantities, including magnitude comparison (e.g., Simmons et al., 2012), ordering (e.g., Finke et al., 2021), transcoding (e.g., Imbo et al., 2014), and number line estimation (e.g., Namkung & Fuchs, 2016). Dual-task studies have demonstrated that processing of symbolic or nonsymbolic magnitudes is affected by a concurrent working memory load (e.g., Maloney et al., 2019; Xenidou-Dervou et al., 2014).

Second, studies have highlighted that inhibitory control skills are implicated in measures of nonsymbolic numerical processing. Most of this evidence concerns performance on dot comparison tasks where control of visual parameters of dot arrays creates congruent trials (where the visual features of the stimuli are positively correlated with numerosity) or incongruent trials (where the visual features of the stimuli are negatively correlated with numerosity). Studies have demonstrated that, when all relevant visual parameters are controlled, congruency effects are observed for accuracy and response time on these tasks by adults and children (e.g., Soltész et al., 2010). Performance on dot comparison tasks is also associated with standard measures of inhibitory control (e.g., Fuhs & McNeil, 2013; Gilmore et al., 2013). It is not yet clear, however, whether this association is an inherent part of nonsymbolic numerical processing itself or is just a consequence of the task (dot comparison) typically used to measure it.

There is also experimental evidence that inhibitory control processes are involved in multi-digit number processing. In a multi-digit comparison task, manipulating the proportion of unit-decade compatible trials (e.g., 86 vs. 52 where 8 > 5 and 6 > 2) compared with unit-decade incompatible trials (e.g., 82 vs. 56 where 8 > 5 but 2 < 6) affects performance in the same manner to manipulating congruencies in cognitive control tasks, for example, Stroop (Macizo & Herrera, 2013).

Third, studies have identified associations between spatial skills and a set of basic mathematical processes. In some cases, these spatial associations are automatic (i.e., spatial-numerical associations; see Cipora et al., 2020, for a review). This includes the well-known SNARC (Spatial Numerical Association of Response Codes) effect where individuals (in cultures with left-to-right reading directions) respond more quickly to larger numbers with the right hand and more quickly to smaller numbers with the left hand (e.g., Wood et al., 2008). Similarly, associations between spatial features and basic processing of single digits are demonstrated by size congruity effects (e.g., Henik & Tzelgov, 1982) and biases in line bisection tasks (e.g., Fischer, 2001).

Beyond these examples of automatic spatial influence on mathematical processes, there is also evidence for a more deliberate influence of spatial skills. For example, number line estimation tasks require individuals to estimate the magnitude of a number by placing it on a line. Spatial skills, including mental rotation (Gunderson et al., 2012; Simms et al., 2016), disembedding (Simms et al., 2016), and spatial orientation skills (Cornu et al., 2017), are associated with children’s concurrent number line task performance and improvements in performance over time (LeFevre et al., 2013). Visuospatial skills are also associated with early patterning skills (Rittle-Johnson et al., 2019) and place-value understanding (Mix et al., 2016).

Finally, there is evidence that language skills also affect basic mathematical processes. Verbal number words constitute one of the three core representations of number (Dehaene, 1992) and thus have a foundational role in mathematical processes. It is consequently not surprising that differences across languages are reflected in children’s early acquisition of number words and concepts. For example, children who speak languages which distinguish between single, dual, and plural, rather than just single and plural, have an advantage in acquiring understanding of the number two (Almoammer et al., 2013; see Sarnecka, 2014, for a review).

The influence of language on mathematical processes is not limited to the early acquisition of number words. The lexical composition of number words continues to influence basic mathematical processing as can be seen by comparing across languages. For example, children learning mathematics in modern Welsh, which has a transparent structure (e.g., 11 is “un deg un” which translates to “one ten one”) were more accurate on two-digit number line tasks than children in matched schools learning in English, which is less transparent (e.g., 11 is “eleven”; Dowker & Roberts, 2015). Similarly, the compatibility effect in multi-digit number comparison is more pronounced for German-speaking than English-speaking adults (Nuerk et al., 2005), which has been linked to the inversion property of German. There are also a complex set of mechanisms by which language may influence place-value (Bahnmueller et al., 2018).

### (e) The connection between general cognitive skills and overall mathematical achievement

As outlined above, there is now a substantial body of evidence that general cognitive skills are associated with proficiency with specific components of mathematics as well as with basic mathematical processes. Alongside this, many studies investigating the role of general cognitive skills have measured overall mathematics achievement (typically using standardised or curriculum assessments), rather than more specific mathematics measures. As we would expect, these studies find evidence that cognitive skills, including working memory (Peng et al., 2016; Spiegel et al., 2021), inhibition (Spiegel et al., 2021), spatial skills (Xie et al., 2020), and language skills (Peng et al., 2020), are associated with mathematics achievement. However, when considered within a multi-level framework, it is unclear whether these relationships arise because these general cognitive skills are associated with either proficiency with specific mathematics components or basic mathematical processes, as described above, or whether there is indeed a separate, direct association between general cognitive skills and overall mathematics achievement. In other words, is there a need within the framework for the connection *e* or is the role of cognitive skills in mathematics achievement entirely explained by connections *a* and *c*?

Why might there be a direct relationship between cognitive skills and mathematics achievement that is not captured by the role of cognitive skills in proficiency with specific components of mathematics or basic mathematical processes? This direct path could exist for two reasons: (1) It may be that cognitive skills are required for the tasks used to measure overall mathematics achievement in a way that is separate from the role of these cognitive skills in the specific components themselves. Measures of mathematical achievement typically include varied items, for example, an item measuring fraction arithmetic might be followed by an item involving reading information from a graph. This requires switching between different topics, knowledge, and skills. Questions will also often be context-based or word problems, requiring participants to build a mental model of the problem situation, identify the mathematical knowledge or skills required, and select an appropriate procedure before carrying this out. These types of activities add cognitive challenges that may not be captured by purer measures of proficiency with specific components of mathematics (e.g., Viterbori et al., 2017). (2) Alternatively, the direct path between cognitive skills and overall mathematics achievement may exist because cognitive skills are needed for classroom learning. To progress in mathematics, children need to pay attention in the classroom, ignore distractions in their environment, and follow instructions from the teacher so they can integrate new knowledge with existing knowledge. These behaviours may place different demands on children’s cognitive skills compared with the role of cognitive skills in specific components of mathematics. These applied executive function skills may be distinct from cognitive executive function processes that are recruited when children actively process mathematical information (Gilmore et al., 2022). Hence, cognitive skills would explain additional variance in mathematics achievement over and above that explained by specific components of mathematics.

We have recently investigated whether there is a separate, direct, relationship between general cognitive skills and overall mathematics in two studies (Cragg, Keeble, et al., 2017; Trickett et al., 2022). In the first study, with children aged 8–9, 11–12, and 13–14 years as well as young adults, we found that specific measures of arithmetic (number fact knowledge, arithmetic procedural skills, conceptual understanding of arithmetic) partially explained the relationship between working memory and mathematics achievement, but a significant direct relationship remained (Cragg, Keeble, et al., 2017). We replicated and extended this in a second study with children aged 12–15 years and incorporating measures of proficiency with a broader range of specific components of mathematics (number fact knowledge, arithmetic procedural skills, conceptual understanding of arithmetic, algebra, and geometry). Again, we found that the specific components of mathematics partially explained the relationship between working memory and mathematics achievement, but a significant direct relationship remained. However, the specific components of mathematics (in particular, number fact knowledge and arithmetic procedural skills) fully explained the relationship between inhibitory control and mathematics achievement (Trickett et al., 2022).

We have shown evidence that a direct path from general cognitive skills to overall mathematics achievement may exist for working memory. It is plausible that it may exist for some cognitive skills but not for others. For example, we might expect language skills to be important for overall mathematics achievement over and above the role of language in proficiency with specific components of mathematics. In contrast, the role of spatial skills in mathematics achievement may be fully accounted for by the role of spatial skills across numerous specific components of mathematics. However, this is yet to be determined.

## What is the impact of mathematical learning experiences?

The process of learning mathematics does not happen in a vacuum, and consequently, we must also consider the impact of children’s mathematical learning experiences. These include informal mathematical experiences (e.g., the Home Mathematics Environment) as well as more formal mathematics education (i.e., the pedagogy and resources used in preschools and schools). The majority of research in mathematical cognition does not take account of these learning activities but considers cognitive (and non-cognitive) factors in isolation of children’s experiences. Mathematics education research has investigated how differences in pedagogy and resources may affect overall mathematics achievement (e.g., Hodgen et al., 2018, 2020) but typically does not consider the impact on lower-level mathematical processes or how they interact with children’s domain-general skills. There is therefore a need for research which considers how different learning experiences affect not only overall mathematics achievement or specific components of mathematics, but also basic mathematical processes, the recruitment of domain-general skills, and the relationships among all of these elements. Below, I give a few examples of research highlighting how learning experiences may affect multiple elements of the framework.

A body of research has investigated children’s understanding of mathematical equivalence. This has demonstrated that children may hold different conceptions of the meaning of the equal sign: children with a *relational* conception understand that the equal sign means that each side of the expression has the same value whereas children with an *operational* conception consider that the equal sign indicates where the answer should be written (Jones et al., 2012; Rittle-Johnson et al., 2011). Children with relational understanding typically have higher overall mathematics achievement and in particular show better learning of algebra (Byrd et al., 2015; Knuth et al., 2006). One reason that children may develop an operational understanding of the equal sign is due to the experiences they have with mathematical material. Many resources (e.g., textbooks, curriculum tests) over-use traditional *operation* *=* *answer* (e.g., 2 + 3 = 5) expressions (Jones et al., 2013; Li et al., 2008), and reliance on these is associated with developing operational conceptions of the equal sign. Changing learning materials to incorporate a wider variety of expression types (e.g., 5 = 2 + 3, 3 + 4 = 2 + 5) can improve children’s conceptual understanding of equivalence and their arithmetic procedural skills (McNeil et al., 2011). This provides an example of how learning experiences (and specifically the resources used) can affect proficiency with specific components of mathematics, that is, conceptual understanding of arithmetic symbols.

Learning experiences may also affect more basic mathematical processes. Considerable interest has been paid to how numbers gain meaning, often referred to as the Symbol Grounding Problem (e.g., Leibovich & Ansari, 2016; Reynvoet & Sasanguie, 2016). Many studies have investigated the magnitude and ordinal properties of symbolic numbers (e.g., De Smedt et al., 2013; Holloway & Ansari, 2009; Lyons et al., 2012; Sasanguie et al., 2017). However, less attention has been paid to the potential influence of children’s informal learning experiences on the development of symbolic number representations. Young children are exposed to varied representations of numbers during the period when they are learning symbolic numbers, and research from artificial learning experiments suggests that the nature of external representations seen may influence the precision of internal numerical representations. Studies with older children learning artificial number symbols suggest that mixed concrete representations (e.g., one cat, two dogs, three cows) are less beneficial than abstract or single concrete representations (e.g., one cat, two cats, three cats; Bennett et al., 2019). This evidence does not directly translate to the situation where young children are learning number concepts and representations; however, it does highlight the potential influence of learning experiences on the development of basic mathematical representations and processes. It is important to study these influences further, particularly given that studies of number books for young children have found that mixed concrete representations are the most common (Ward et al., 2017).

The impact of learning experiences may not be the same for all children but may instead interact with child-level factors. For example, research has highlighted that individuals differ in their spontaneous attention to numerical or mathematical aspects of their environment (e.g., Spontaneous Focusing on Numerosity [SFON], also referred to as Attention to Number [ATN]; Batchelor et al., 2015; Chan & Mazzocco, 2017; Hannula & Lehtinen, 2005; Rathé et al., 2016). While some studies have identified that these tendencies are associated with concurrent and future mathematical achievement (see reviews by McMullen, Chan, et al., 2019; Rathé et al., 2016), it remains to be determined whether this is best conceived of as a separate domain-specific (i.e., mathematical) process (or arising from other domain-specific processes) or whether it reflects a more general perceptual or attentional process. Currently, little is known about the relationship or interaction between these SFON/ATN processes and different learning experiences (although see McMullen, Hannula-Sormunen, et al., 2019). Identifying the nature of these differences in dispositional tendencies would provide a clearer picture of whether and how they represent causal relationships with mathematics achievement and learning.

Learning and cultural experiences may also affect the influence of general cognitive skills on basic mathematical processes. Evidence of spatial influences on basic numerical processing (i.e., spatial-numerical associations, Cipora et al., 2020) was outlined above. However, the nature of these associations are culturally influenced. For example, spatial-numerical associations may be reduced or the direction changed for individuals who write right-to-left or top-to-bottom (Toomarian & Hubbard, 2018). Differences in the spatial-numerical gestures used by parents (McCrink et al., 2018) and cultural variation in the nature of finger counting (Bender & Beller, 2012) highlight how learning experiences may affect the nature of basic mathematical processes and how domain-general cognitive skills may be recruited to underpin these.

One area where considerable attention has been paid to the nature of learning experiences is in relation to Cognitive Load Theory (Sweller, 2010). A large body of literature has investigated how modifications to educational tasks and the support provided to children can affect specific components of mathematics by modifying the demands of the tasks (e.g., decreasing load on working memory). For example, studying worked examples, where the steps to solve a problem are broken down and demonstrated to learners, can lead to improved learning of the target material (see Perry et al., 2021, for a review). Considering this research within the framework highlights the importance of considering multiple levels of mathematics. Approaches such as worked examples or providing schematic prompts may improve proficiency with one specific component of mathematics (e.g., arithmetic procedural skills), but we also need to consider the impact of these approaches on other components of mathematics (e.g., conceptual understanding or problem solving) as well as overall mathematics achievement.

## What do we still need to know?

As outlined above, there is existing evidence to support some aspects of the proposed framework. However, several important questions remain.

### What are the most important basic mathematical processes and specific components of mathematics that are necessary to understand the mechanisms of mathematical cognition?

Table 1 lists a set of candidate basic mathematical processes and specific mathematics components that have been the focus of previous research. This list is not exhaustive, and an important task for the field is to investigate which basic mathematical processes are essential for higher-level mathematics performance and which specific components of mathematics form coherent elements of knowledge, skills, and understanding. Identifying which are the most important basic mathematical processes and which are the most important specific components of mathematics is essential to develop theories incorporating causal mechanisms of learning, as well as to inform mathematics education. If children have difficulties with these processes and components, then they are more likely to struggle with mathematics. Consequently, learning activities should focus on these processes and components, so we are confident that children are able to acquire or develop them securely.

The framework as presented includes a three-level hierarchy whereby basic mathematical processes are recruited in the performance of specific components of mathematics, which in turn combine to give rise to an individuals’ overall level of mathematics achievement. However, it is likely that, in some sub-domains at least, multiple levels of increasing broad components may be identified. Moreover, these basic processes and components may change over development. For example, for children, place-value understanding may lie at a somewhat higher level of the hierarchy, which arises out of a combination of lower-level mathematical ideas and processes. However, with increasing experience, place-value understanding may become more automatised and serve as an input to higher levels of understanding. What is important for the development of improved theoretical models is that we are careful to use the right level of specificity/generality to answer the questions of interest.

### How “specific” are the basic domain-specific mathematical processes and how “general” are the domain-general cognitive skills?

The question of how mathematically or numerically specific basic mathematical processes are has already attracted considerable attention. Most notably, this has been debated in relation to magnitude representations (i.e., the Approximate Number System (ANS) or analogue magnitudes). Under some proposals, the ANS involves specifically numerical representations and processes (Clarke & Beck, 2021; Feigenson et al., 2004; Whalen et al., 1999). However, evidence of the influence of non-numerical visual characteristics on non-symbolic task processing (Gebuis & Reynvoet, 2012; Szűcs et al., 2013) has led others to propose that performance on these tasks in fact involves generalised magnitude representations and relies on general cognitive processes (e.g., Leibovich et al., 2016; Walsh, 2003; often preferring the term Approximate Magnitude System). This remains a question of active research. Similarly, the numerical specificity of order processing mechanisms that are related to mathematics outcomes has also been debated with evidence that more general order processing skills underlie the relationship between numerical order processing and mathematics skills (Morsanyi et al., 2017). This casts doubt on the role of order processing as a basic specific mathematical process. Finally, recent increased attention to patterning as an important skill related to mathematics outcomes (Rittle-Johnson et al., 2019; Wijns et al., 2019) invites similar questions. The extent to which patterning skills can be identified as a specific mathematical skill separate to more general analogical reasoning skills remains to be determined (Kidd et al., 2014; Zippert et al., 2019).

A parallel question concerns how “general” are domain-general cognitive skills. This has been particularly questioned in relation to executive function skills (Raghubar et al., 2010). Studies have investigated whether the relationship between executive function skills and mathematics outcomes is stronger when executive function tasks involve numerical or mathematically relevant information rather than non-mathematical information. The evidence here is mixed, with some studies finding a stronger relationship for mathematical content (e.g., Bull & Scerif, 2001) although meta-analyses find no moderating effect of domain-specificity for working memory (Peng et al., 2016). There is some evidence from experimental paradigms that domain-general executive function processes can transfer to mathematical processes (Eaves, Gilmore, & Cragg, 2022). However, this transfer effect does not itself indicate that the cognitive control processes involved in mathematical processing are “content free” general processes that are common across all domains.

Overall, it may be that the basic cognitive processes involved in mathematics are better conceived of as a continuum between more general and more specific, rather than a dichotomy between domain-general and domain-specific.

### What is the role of affective factors?

A major omission from the framework as presented here is where affective factors fit. It is very well established that individuals’ emotions and attitudes to mathematics are associated with mathematics outcomes (Goetz et al., 2007; Pinxten et al., 2014). Most notably, research on mathematics anxiety has identified that this has a pervasive influence across many domains (Cipora et al., 2022; Dowker et al., 2016). However, the mechanisms underlying this relationship are as yet unclear (Carey et al., 2016). While there is consistent evidence that mathematics anxiety is associated with overall mathematics achievement and proficiency with specific components of mathematics at the higher levels of the framework (Barroso et al., 2021; Zhang et al., 2019), it is not yet clear whether there is an association with more basic mathematics processes (Dietrich et al., 2015; Maloney et al., 2011).

Theories of mathematics anxiety often implicate working memory and attention as a mechanism for the impact on mathematics achievement (Ashcraft & Kirk, 2001), highlighting that mathematics anxiety may affect the link between cognitive skills and mathematics outcomes. Furthermore, mathematics anxiety can lead individuals to avoid mathematical activities (Ashcraft, 2002). Therefore, mathematics anxiety may also affect the influence of learning experiences on mathematics learning. Consequently, it may be that affective factors such as mathematics anxiety are best conceived of as layered across the whole of the framework, rather than an influence on specific elements or relationships within the framework. Further research is needed to determine the multiple mechanisms by which mathematics anxiety and other affective factors (e.g., self-concept, motivation) are associated with mathematics performance and behaviours.

### Is there a direct pathway between basic mathematical processes and mathematics achievement?

As described above, the framework currently proposes a direct pathway between general cognitive skills and overall mathematics achievement that is not accounted for by the involvement of these general cognitive skills in specific components of mathematics. In contrast, there is no direct pathway from basic mathematical processes to overall mathematics achievement. It is assumed that the involvement of basic mathematical processes in overall mathematics achievement is entirely accounted for by their role in specific mathematics components. While many studies have investigated basic mathematical processes and either overall mathematics achievement or proficiency with specific components of mathematics (e.g., arithmetic procedures), only a smaller number of studies have investigated all three (e.g., Vanbinst et al., 2012; Wang et al., 2016). Among those that have, there is evidence that proficiency with specific components of mathematics explains the relationships between basic mathematical processes and overall mathematics achievement. For example, symbolic number knowledge explains the relationship between nonsymbolic arithmetic and overall mathematics achievement (Gilmore et al., 2010), and count sequence knowledge explains the relationship between numerical order processing and overall mathematics achievement (Gilmore & Batchelor, 2021). Similarly, Douglas et al. (2020) found that numerical order processing was no longer associated with higher-level measures of fraction and algebra performance once arithmetic skills were accounted for. Rebholz et al. (2022) found that the relationship between number line estimation and mathematics achievement was partially mediated by arithmetic fluency (and fully mediated by arithmetic fluency and visuospatial skills). Future research should include measures of proficiency with specific components of mathematics as well as overall mathematics achievement to establish whether there is evidence for a direct pathway between basic mathematical processes and mathematics achievement and to consider plausible mechanisms that could underlie such a relationship.

## What are the implications of the framework?

As described above, the proposed framework is not intended as a model of mathematical processing, but rather a structure to make sense of existing empirical evidence, identify important unanswered questions, and give structure to existing models of mathematical cognition. However, a number of implications relating to mathematical processing do arise from the framework.

A first implication is that overall mathematics achievement arises from a complex interaction of cognitive and non-cognitive influences. Consequently, we would expect differences in mathematics achievement to arise from an interaction of lower-level skills and processes, and not just from the simple additive effects of a set of more basic components. In other words, the framework would be falsified if we found that no more variance in individual differences in mathematics were explained by conceptualising mathematics in this multi-level framework than by simply considering the additive effects of a list of lower-level skills.

Some studies have begun to explore how far interactions between cognitive (and to a lesser extent non-cognitive) factors explain variance in overall mathematics achievement. We found that variance in 5- to 6-year-old children’s mathematics achievement was explained by the interaction between children’s procedural arithmetic skills, conceptual understanding of arithmetic, and working memory over and above the individual variance associated with each of these components and skills (Gilmore et al., 2017). Similarly, Ribner et al. (2017) found that children’s mathematical learning was predicted by the interaction between executive function skills and early mathematical skills.

Second, I hope that an important contribution of the framework is to identify that we need more work (empirical and theoretical) focusing on mechanisms, rather than just associations. These mechanisms form the links between elements of the framework. We now have considerable evidence of associations between individual differences in basic mathematics processes or general cognitive processes and individual differences in proficiency with specific components of mathematics or overall mathematics achievement. However, less work has focused on identifying whether these associations represent causal mechanisms, or the nature of any such mechanisms. The multi-level framework may be helpful in identifying the nature of the mechanisms that need investigation. For example, it would be less helpful to think about the mechanisms that link magnitude comparison skills or working memory with overall mathematics achievement than to focus on more specific mechanisms, for example, the involvement of magnitude comparison skills in selecting arithmetic strategies (e.g., Vanbinst et al., 2012) or the involvement of working memory in decomposition strategies in mental arithmetic (e.g., Imbo et al., 2005).

A third important implication concerns methodology. We need to think more carefully about the specific element of mathematics that we wish to measure in any given study. This decision should be driven by consideration of the aims of the studies. Sometimes (e.g., when considering the factors that influence children’s progress through education) using an overall measure of mathematics achievement is appropriate, but at other times (e.g., when exploring causal mechanisms), we need to focus on more precise measures of proficiency with specific components of mathematics. Importantly, what should not drive decisions about the use of measures is the simple ease of the measure involved. Different measures of mathematics outcomes are not all equivalent, and therefore we should not use an arithmetic fluency measure just because it is quick and easy to administer unless we are specifically interested in measuring arithmetic fluency. To select appropriate measures, we need to identify more clearly what skills and knowledge different mathematical tasks measure and not conflate tasks with constructs.

Fourth, the framework also provides a structure for consideration of mathematical learning difficulties. The complexity of the components involved highlight that mathematical difficulties can have multiple contributory factors. These may arise in part from difficulties with basic mathematical processes (e.g., magnitude comparison or order processing); however, there are many potential contributory factors (Bartelet et al., 2014; De Smedt, 2022).

Finally, the framework contributes to debates about pedagogical decisions. The framework highlights that individual differences in mathematical achievement arise out of a complex interaction of multiple cognitive and non-cognitive skills influenced by learning experiences. Consequently, pedagogy should not focus on some components (e.g., fluency with number facts) at the cost of others (e.g., conceptual understanding). Mathematical cognition research can contribute to this debate by identifying the basic mathematical processes and specific components of mathematics that are most important for overall mathematics achievement and learning and by demonstrating how different learning experiences affect their development. This information can support policymakers and curriculum designers in ensuring that children receive broad and rich learning experiences that target the skills and understanding that they need.

## Conclusion

A complex set of domain-general and domain-specific skills are involved in mathematical learning and performance. To understand the mechanisms and processes involved, we need to recognise that mathematics is not a single construct but involves multiple components which draw on multiple basic processes. Mathematical cognition research has flourished over the past two decades, but before this research can have a positive impact on education in the classroom, we need to recognise the complexity of mathematics and to identify how children’s experiences influence learning.
